# Stakeholder perspectives on the effects of environmental and socio-economic factors on children's health and learning: a qualitative study in Greater Manchester, England

**DOI:** 10.3389/fpubh.2025.1550439

**Published:** 2025-06-18

**Authors:** Sarah Daniels, Hua Wei, Anne Clayson, Martie van Tongeren, Thomas Bannan, Melanie Carder, Luke Munford, Nicola Gartland

**Affiliations:** ^1^Division of Population Health, Health Services Research and Primary Care, School of Health Sciences, Faculty of Biology, Medicine and Health, The University of Manchester, Manchester, United Kingdom; ^2^Department of Earth and Environmental Sciences, School of Natural Sciences, Faculty of Science and Engineering, The University of Manchester, Manchester, United Kingdom

**Keywords:** air pollution, air quality, children's health, cognitive development, qualitative study

## Abstract

**Introduction:**

Environmental factors such as poor air quality may exacerbate health inequalities among children. This study aimed to explore stakeholders' views on the impact of environmental and socio-economic factors on children's health and learning, and the effectiveness of local air quality initiatives.

**Methods:**

Between April and June 2024, we conducted 15 semi-structured interviews with primary school teachers, local government and transport representatives from Greater Manchester (GM), a city region with high levels of deprivation in the Northwest of England. Inductive thematic analysis was completed using NVivo14.

**Results:**

Four key themes were identified: (1) health and developmental concerns for GM primary school children, (2) factors associated with children's health and development, (3) ongoing initiatives to improve air quality in and around schools, including whether and how such initiatives were evaluated, and the perceived effectiveness, barriers and facilitators of the initiatives, (4) key priorities for future research. Concerns over children's health and development including children's learning, road safety, physical health, attendance, and mental wellbeing were frequently expressed. Participant views about air quality as a contributing factor to children's health and development were mixed. Participants also expressed concern over socio-economic factors affecting children's health and development, including deprivation, housing conditions, and access to green spaces. The identified air quality initiatives mainly targeted traffic reduction and active travel, but evaluation of initiatives faced challenges, particularly time constraints hindering data collection, and there were mixed opinions on effectiveness. Barriers to implementation included parental resistance, busy schedules and road safety concerns. Community engagement and involvement of children were seen as facilitators, but funding and sustained local government support were challenges. The rise in Special Educational Needs (SEN) and cognitive issues, particularly evident post-COVID, and the role of environmental factors was considered as a gap in knowledge.

**Conclusion:**

This study highlights the complex relationship between air pollution, socio-economic disparities, and children's health in GM. Inequitable resources and behavioral resistance hinder progress, but stronger stakeholder collaboration and evidence-based strategies can help. The post-COVID rise in SEN and learning difficulties calls for research. Future studies should adopt multidisciplinary, longitudinal approaches to assess the long-term impact of air quality initiatives.

## 1 Introduction

Air pollution is a widespread health concern for everyone but poses a significant risk to children due to their increased vulnerability (1). Exposure at an early age to harmful air pollutants, such as particulate matter (PM), nitrogen dioxide (NO_2_), and ozone (O_3_), can lead to reduced lung function and increase the likelihood of acute respiratory illnesses and asthma in children (2, 3). These pollutants are emitted from various sources, including vehicle exhausts, industrial emissions, agriculture, domestic wood and coal fires, and chemical solvents (4). Children have higher breathing rates, immature immune systems, developing lung function, and tend to have active play habits and more time spent outdoors which means they are at increased risk of disease from air pollution (2).

Beyond just affecting their respiratory health, it has also been shown that air pollution significantly affects the developing central nervous system (5). Ultrafine PM (airborne particles that are <0.1 mm in diameter) may affect the nervous system directly through crossing the olfactory bulb and blood brain barrier, contributing to neuroinflammation and cell loss (6). The blood-brain barrier has been observed to be more permeable during childhood than later in life, increasing the likelihood of ingested and inhaled pollutants entering the brain, causing inflammation of tissue, and further damaging vulnerable brain regions (6).

There is growing evidence that exposure to air pollution can affect children's neurological and cognitive development (7–10). These effects have significant impacts on learning and education outcomes/attainment over time (11). Evidence from studies, primarily from outside the UK, suggests that both short- and long-term exposure to traffic-related air pollution (TRAP) impedes the developmental trajectory of cognitive function in children, particularly executive functions such as working memory and attention (12). Several studies, particularly those from the BREATHE project, demonstrate the long-term effects of air pollution, with exposure over a 1- to 3.5-year period leading to slower cognitive development compared to children in lower-pollution areas (13, 14). Studies also show that short-term exposure—such as pollution levels measured on test days—can negatively impact academic performance, such as in reading and maths test scores (15). While evidence suggests that higher levels of air pollution negatively impact cognitive development compared to lower pollution levels (14, 16), research on the effects of relocation to less polluted areas remain limited, particularly regarding its long-term influence on cognitive recovery in children (7, 17).

Given that children are especially vulnerable to the negative impacts of air pollution, reducing pollution levels during the journey to and from school and within school environments could offer significant health benefits. A 2024 study showed that 86% of 147 new school sites in England exceeded all three World Health Organization (WHO) global air quality targets for PM_2.5_, PM_10_, and NO_2_, with every location surpassing at least one (18). Schools in Greater Manchester (GM) were reported to have some of the highest pollution levels in the UK (18). Whilst long term trends show that there has been an improvement in air quality for NO_2_, PM_10_, and PM_2.5_ data, the Greater Manchester Combined Authority (GMCA) has implemented a Clean Air Plan to reduce NO_2_ levels on local roads to meet legal standards (19). This plan also focuses on enhancing air quality for schoolchildren by encouraging active travel, such as walking and cycling, and offering resources to schools to tackle pollution (20).

Deprivation in UK cities is closely linked with poor air quality. In areas of high deprivation, such as GM, where one in three children live in poverty (21), children are more likely to be exposed to higher levels of air pollution due to proximity to traffic and industrial activities (22, 23). Moreover, socio-economic disadvantage among children is also linked to worse outcomes in cognition and academic performance (24–28). The combined effects of poor air quality and deprivation may amplify existing health inequalities among children and have lifelong impacts on their health and labor market involvement.

Before conducting semi-structured interviews with local key stakeholders about air pollution and its impact on children, we scoped both academic and gray literature to identify school initiatives implemented in GM to tackle air pollution. Our review found that in GM, several travel-related initiatives have been introduced in primary schools (attended by children aged 5 to 11 in England) to address this issue; however, we only identified evidence from gray literature, with no relevant academic studies found in the peer-reviewed literature. The School Streets program, for example, supported by Transport for Greater Manchester (TfGM) and the Government's Active Travel Fund, limits vehicle access during school drop-off and pick-up times, promoting safer, traffic-free zones (29, 30). The Living Streets WOW (Walk Once a Week) initiative encourages children to travel actively to school using an interactive travel tracker and is funded by a £60 million package from Active Travel England (31). Various cycling initiatives, such as Bikeability, a national UK program, teach both children and adults safe cycling skills and road knowledge (32). The Park and Stride scheme, part of the Living Streets program, encourages families living too far from school to park a short distance away and walk the remaining distance (33). Additionally, the TfGM-funded Modeshift STARS scheme offers GM schools the opportunity to create travel plans for free, set goals like increasing scooter and cycle storage, and receive accreditation based on their achievements (34). Small scale research initiatives were also implemented in some areas of GM. For example, the installation of green screens in four primary schools in GM has shown to decrease airborne PM concentrations (35).

One example of an indoor air quality initiative is the use of air purifiers in schools. Scientific studies examining the implementation of air purifiers in GM schools are limited but Thomas et al. conducted air quality measurements in six schools to evaluate the effectiveness of High Efficiency Particulate Air (HEPA) filters in reducing indoor pollutant levels (36). The results showed that HEPA purifiers were effective in controlled settings, but their real-world efficacy varies. A 37.4% PM_2.5_ reduction was observed at times, though overall results were inconclusive due to high air exchange rates with outdoor or adjacent rooms, and poor compliance with experimental protocols in schools (36).

Socio-economic factors can directly influence children's health and learning outcomes (24, 26, 27). Nevertheless, the interplay between deprivation and air pollution highlights how socio-economic disadvantages heighten exposure to harmful pollutants (37, 38), which has been linked to cognitive impairments across various populations (39). Moreover, recent findings suggest that access to nature can play a crucial role in supporting cognitive functioning in children, further emphasizing the multidimensional nature of these environmental and social determinants (40). Understanding the complexities of how these factors interact in their impact on children's cognitive development (with each other, as well as with a myriad of other factors) requires a holistic approach that incorporates the priorities of the community and local authorities. The study aimed to explore the various effects of air pollution on outcomes for children and investigate what air quality initiatives were being implemented in and around primary schools in GM, to gather stakeholders' views on these initiatives, to explore their perceived effectiveness, and the barriers and facilitators influencing their success.

## 2 Methods

The overall study design involved interviews with primary school staff, and GMCA and TfGM representatives. Semi-structured interviews were selected to allow flexibility to explore specific themes and enable participants to provide detailed and comprehensive responses (41).

We have followed the Consolidated Criteria for Reporting Qualitative Studies (COREQ) to report the methods and findings (42). A checklist can be found in Supplementary File 1.

The study obtained ethical approval from The University of Manchester Research Ethics Committee.

### 2.1 Recruitment and data collection

We utilized multiple methods to advertise the project and facilitate participant recruitment, including reaching out to potential participants by direct contact (e.g., publicly available work email address or phone number), presenting at community events and through established networks. A flier was created and circulated at community events and through existing contacts during the later stages of recruitment to increase interest.

Participants received a Participant Information Sheet at least 24 h before the interview. Consent was obtained either verbally before the commencement of the interview or through the signing of a consent form. The interviews were audio recorded and carried out either online via Zoom or Microsoft Teams teleconferencing platforms, or in-person. Interviewing researchers also took field notes. Three trained researchers with extensive experience in qualitative research (HW, SD and NG) conducted all interviews, with most interviews conducted by two researchers.

We followed purposive sampling and selected schoolteachers for their daily interactions with children and understanding of how the school environment impacts health and learning. Primary schools were chosen as they are critical stages of rapid development, allowing early identification of health challenges. GMCA and TfGM were included for their active role in air quality initiatives and community engagement, such as the Clean Air Plan. We stopped recruitment to the study when the research team agreed that data saturation had been reached, as no significant new information or themes were identified during the later stages of the interviews.

The interviews began with open-ended questions aimed at exploring participants' awareness and involvement in air quality initiatives. These questions encouraged discussion about the effectiveness of such initiatives, alongside the barriers and facilitators influencing them. All questions were asked without the researchers providing any prior information on the initiatives. The questions also addressed concerns about sources of air pollution, its effects on children's health, and the vulnerability of specific groups. A key aim was to assess the role of socio-economic factors in shaping these issues. We also outlined our future research proposal on investigating air pollution, air quality, and children's cognitive development, to seek their feedback and suggestions. The interview schedules are provided in Supplementary File 2.

Interview audio recordings were anonymized and transcribed by a university-approved transcription service.

### 2.2 Data analysis

Inductive thematic analysis was performed using NVivo14 software (43, 44). An initial codebook was developed by having two coders (HW and SD) independently code three transcripts. The related codes were combined to generate developing themes and sub-themes that reflect the underlying ideas or concepts in the data set. Emerging themes were reviewed by HW and SD and refined to ensure they accurately represent the data. The initial codebook was reviewed and discussed with the wider research team leading to an iterative re-coding process. This continued until the team reached a consensus on the final coding structure. To evaluate inter-coder reliability, the coders' work on the three transcripts was combined, yielding a high agreement rate of 99% and an average kappa coefficient of 0.65, which falls within the “good” range for strength of agreement (0.61–0.80) (45). Following this, one researcher independently coded the remaining transcripts. The finalized codebook can be found in Supplementary File 3.

While the interview questions were framed around air quality, socio-economic factors, related initiatives and children's cognitive development, the semi-structured approach allowed for flexibility and open for new themes to be identified, enabling participants to highlight topics they considered significant, even if these were not initially prioritized by the researchers (46).

## 3 Results

Between early April and mid-June 2024, we conducted 15 interviews. Ten interviews with headteachers or senior leadership teams of primary schools, three interviews with representatives from GMCA and two from TfGM. Each interview lasted 30–60 min. TfGM and GMCA representatives included roles in relation to environment, public health and active travel. The primary schools and participants were located across various areas of GM, including Manchester, a major urban city and economic hub; Oldham, a former industrial town with suburban areas and ongoing regeneration; Stockport, a suburban town with easy access to Manchester; Tameside, a mix of suburban and industrial areas with regeneration; and Salford, an urban city with significant redevelopment. See Table 1 for participant characteristics.

**Table 1:** Participant characteristics.

**Interview reference**	**Stakeholder type**	**Number of participants**	**Role/responsibility**	**Local authority**
P1	City council	1	Education and environment	Manchester
P2	Primary school	1	Senior leadership	Manchester
P3	TfGM	1	Transport	GMCA
P4	Primary school	1	Senior leadership	Stockport
P5	City council	2	Environment and transport	Salford
P6	Primary school	1	Teacher	Manchester
P7	TfGM	1	Environment	GMCA
P8	City council	2	Public health	Manchester
P9	Primary school	1	Teacher	Stockport
P10	Primary school	1	Senior leadership	Manchester
P11	Primary school	1	Teacher	Tameside
P12	Primary school	1	Senior leadership	Manchester
P13	Primary school	2	Senior leadership and teacher	Manchester
P14	Primary school	1	Teacher	Oldham
P15	Primary school	1	Senior leadership	Tameside

GMCA, Greater Manchester Combined Authority; TfGM, Transport for Greater Manchester.

Four overarching themes were identified: (1) health and developmental concerns for primary school children in GM, (2) factors associated with children's health and development, (3) ongoing initiatives to improve air quality in and around schools, including whether and how such initiatives were evaluated, and the perceived effectiveness, barriers and facilitators of the initiatives, (4) key priorities for future research.

### 3.1 Main health and developmental concerns for primary school children in GM

Participants (P2, P4, P6-7, P9-14) expressed that there were ongoing concerns including learning ability, road safety, physical health, attendance and mental health when considering primary school children's health and development. These concerns were raised based on general observations rather than specific environmental factors.

A range of learning difficulties were discussed included reading, concentration, memory, autistic spectrum disorders and attention deficit hyperactivity disorder (ADHD). Some interviewees highlighted high rates of children with special educational needs (SEN) in late years as an indication of the emerging problem. For example,

“*We certainly have significant children with issues, with cognitive delay, speech and language issues. I know that's not quite related but, like, far higher rates of asthma, far higher rates of SEN than… Our SENs numbers, Ofsted [Office for Standards in Education] don't believe our SEN numbers.”* (P2)

Participants also highlighted road safety as a major concern. Several teachers noted that schools were often adjacent to or surrounded by busy roads (P2, P4, P9-10, P14). Additionally, there were concerns about the lack of safe crossings (P2, P12) or that existing crossings were not ideally located (P2). P2 mentioned that a school crossing patrol guard was badly injured due to a poorly placed crossing. They noted that drivers often speed up after exiting a roundabout, close to where the crossing is located. They said that “*I think a child would have died. It was really bad*.” They also noted that another crossing is situated inconveniently halfway down a main road, discouraging pedestrian use.

Concerns over physical health issues were also raised by the participants, specifically in relation to asthma (P2, P4, P6-7, P11-14), weight issues or being less active (P6, P13) and allergies (P14). Attendance was highlighted as a concern. P12 noted that while the school has traditionally exceeded national attendance statistics, attendance has become a more significant issue since COVID-19, reflecting a national trend. The participant noted that parents are now more cautious about sending their children to school when they are unwell. However, they highlighted the need for fostering greater resilience in encouraging attendance. Another attendance issue is extended family visits abroad during school term time (P11-12). The mental health issues raised by participants included Year 6 students, who were in Year 3 during COVID, lacking the expected social skills for their age, appearing immature and struggling with relationships (P2). They were considered not as socially mature as previous Year 6 cohorts.

### 3.2 Main factors related to the concerns

Participants mentioned environmental, socio-economic, and lifestyle influences—such as diet, vaccines, educational challenges, and family transience—as key contributors to the identified concerns about children's health and development.

#### 3.2.1 Environmental factors

The most common environmental factors discussed were TRAP-related or indirect effects of traffic, including car emissions (P1, P4-5, P7-10, P15), traffic diversions (P2), noise (P2), and vibration (P2).

“*I think the main one, certainly the one that springs to mind immediately, is the carbon dioxide from car exhausts.”* (P1)

“*So we decided to go down the line of the school street because the parking, traffic, the congestion, the fumes and the danger to the children was immense.”* (P4)

Local environmental factors, such as wood burning (P1, P5), increased household emissions from heating in densely packed terraced housing or household items (e.g., Yankee candles, deodorants, hairspray etc.) (P11, P7), and littering or fly-tipping (P13) were also mentioned. One participant (P2) believed that the building design might have contributed to high air pollution in and around the school as part of the school was below road level. Another participant (P4) mentioned that during the COVID-19 pandemic, it was mandatory that all doors and windows must be open and that let in TRAP. Global emissions such as volcanic ash from Iceland was also considered an environmental factor (P7). Participants reported that heatwaves resulted in very high temperatures in the classrooms (P4, P11) and prevented children from going out in the summer (P2).

Notably, three participants expressed no concern about air quality in and around the schools (P10, P12, P14).

“*So for us this [air pollution] is not something that I would ever really consider as an issue… I think these children are very lucky. They've got such lovely fresh air, lovely outdoor spaces. We have a forest school, we have a pond, and there's just lots of outdoor opportunities.”* (P14)

Some participants concerned about rising asthma and allergies in children were unsure or unconvinced of environmental causes (P4, P7, P14).

“*If you look at the asthma levels in the UK, they're one of the highest, they're up there with Portugal, of highest in Europe. Yet, the pollution levels are some of the lowest.”* (P7)

A minority of participants remarked that air pollution may contribute to cognitive development (P9, P4) and SEN (P13). Though participants also felt that more knowledge was needed to confirm a link between air quality and cognitive development (P4, P15).

#### 3.2.2 Socio-economic factors

Multiple socio-economic factors were considered to impact children's health and development. The participants talked about deprivation in GM in general and acknowledged multiple dimensions of the phenomenon, as one participant stated when asked about the role deprivation has on the effects of air pollution within the borough:

“*Is deprivation seen through a lens of income, is it seen through a lens of transport, is it seen through a lens of stress and other mental health factors?”* (P8)

Geographical disparities were perceived, with Central Manchester seen as deprived and South Manchester as affluent. For example, when asked about whether deprivation or socioeconomic disparity has an effect on air pollution in their borough, P1 highlighted housing conditions in deprived central areas as more crowded, with poor air quality due to nearby industrial activities, substandard building quality, and lack of green spaces. Two participants from a primary school in a deprived area, expressed that the area was missing out on initiatives related to active travel and green spaces, compared to wealthier areas where local environmental activists played a role to facilitate such initiatives (P2). This sentiment was echoed by P1 who also felt that parents from affluent areas were more likely to work from home, and more often advocate against car use for school drop-offs. As P1 stated:

“*[Area in GM] is the most affluent ward in the city, and at the same time, contributes the highest levels of emissions, but probably has some of the highest levels of environmental activism, in the city, at the same time. And they're in quite a privileged position to be able to say, people shouldn't be using their cars to drop off the children, because, I think there's something around, they have the highest levels of people who can work from home.”* (P1)

P1 suggested environmental activism might overlook the difficulties faced by those in manual labor jobs, who cannot work remotely and depend on cars for commuting and dropping their children at school. Furthermore, poorer city-center areas were perceived to have high levels of vehicle traffic coming in from wealthiest areas, as people travel into Central Manchester (P2). In response to questions about whether some children are more vulnerable to the effects of air pollution and air quality than others, or the follow-up question on the role of deprivation and socioeconomic disparity in these effects, some participants expressed the view that deprived areas are often located near main roads and are therefore more affected by TRAP (P4-5, P15). One participant noted that indoor air quality in these homes was often worse due to household smoking (P9).

Diverse social demographic background, transient families (e.g., asylum seekers and refugees) and language barriers were among the socio-economic factors that participants perceived as impacting children's learning development (P2, P9, P11-12). These factors were mentioned spontaneously by participants, without being prompted by questions specifically about the effects of deprivation and socio-economic disparity on children's health and development. Minority and low income communities were considered to be disadvantaged when it comes to maintaining a healthy immune system and overall health (P7). For example, P7 independently expressed that COVID-19 disproportionately affected Black communities due to vitamin D deficiency during lockdowns as this is important for immune support. Additionally, P7 perceived those individuals with both limited finances and poor diets, were significantly disadvantaged.

#### 3.2.3 Other factors

Participants openly discussed various factors affecting children's health and education, influenced by community attitudes and lifestyle choices. For example, it was noted that some communities place high importance on cars as a status symbol which results in children being driven around rather than walking (P2). Participants discussed the role of diet and antioxidants in mitigating pollution, potential links between dietary factors, like gluten, and cognitive conditions, and the importance of sleep, hydration, and healthy living (P7, P12). There was concern about the number of vaccines children receive and their effects on health (P7). The participants also mentioned educational challenges, including the need for schools to adapt teaching methods to accommodate diverse learning needs and the prevalence of SEN among children (P11, P14). The participants also touched on the transient nature of some families, particularly parents who are educated abroad, and the challenges faced by children who move frequently (P2).

### 3.3 Air pollution and air quality initiatives

Table 2 summarizes the air quality initiatives implemented in and around primary schools in GM that were mentioned by the study participants. These include initiatives like School Streets, digital trackers such as WOW, cycling initiatives, and the use of air purifiers and air quality monitors in classrooms and outdoors. These measures were highlighted during interviews, where participants shared their awareness of existing initiatives.

**Table 2:** School initiatives in GM.

**Type of initiative**	**Summary of the key initiatives discussed by participants:**	**Highlighted in interview (participant reference number)**
School commute	**1. School streets**: Temporarily restricts motor vehicle access around schools during peak times to create safer environments, supported by local councils and volunteers.	P1, P3-4, P7-8, P14-15
	**2. Living streets WOW**: Encourages children to walk, cycle, or scoot to school, funded by Active Travel England, promoting at least weekly active travel.	P2-3, P5-6, P15
	**3. Parking and anti-idling enforcement**: Involves PCSOs and CEOs enforcing parking rules and addressing idling near schools, with children trained as junior PCSOs. Anti-idling and anti-parking signage and barriers.	P6, P8, P12-13
	**4. Cycling initiatives**: Includes Bikeability lessons for safe cycling, grants for cycle storage, cycle path improvements, and a Bike Library that lends bicycles to children and parents.	P3, P5, P10, P13, P15
	**5. Park and stride**: Encourages families to park a short distance from school and walk the rest, part of the Living Streets program.	P5-6, P12
	**6. Modeshift STARS:** schools to develop free travel plans aimed to increase active travel, earning accreditation for successful implementation.	P1, P3, P15
	**8. Educational efforts**: Schools promote active travel as healthy and eco-friendly through class discussions, walking initiatives, and train trips.	P9, P11, P13
	**9. Safe routes**: Parent Walking Buses, construction of zebra crossings.	P1, P12
School environment	**1. Indoor air quality**: Installation of air quality monitoring systems in classrooms and air purifiers during the COVID-19 pandemic to maintain a cleaner learning atmosphere.	P1-2, P6, P8, P14-15
	**2. Green screens**: Installation of green screens to capture vehicle emissions.	P1-2, P4, P8, P15
	**3. Other nature-based solutions**: Beautification of walking/cycle paths to encourage active travel, National Education and Nature Park project.	P1-2, P13
	**4. Educational efforts**: Grow your own' planting, Zero Carbon Schools curriculum in classroom discussions	P13
	**5. Outdoor air quality**: monitoring, car park closure.	P1, P15

CEOs, Civil Enforcement Officers; GM, Greater Manchester; PCSOs, Police Community Support Officers; WOW, Walk Once a Week.

#### 3.3.1 Evaluation and effectiveness of initiatives

Participants reported evaluations using the Modeshift platform and WOW digital tracker, or other trackers, for initiatives such as Living Streets or School streets (P1-3, P5-6, P13, P15). Both platforms assessed the travel modes used by children to get to school through hands-up surveys conducted in class or through self-reporting (P1, P3, P10). However, P1 stated that teachers have reported a discrepancy between pupils' observed behavior and their survey responses, noting some pupils who arrive at school by car claim they cycled, likely to appear more eco-friendly (P1). Furthermore, participants felt there was a lack of GM wide evaluation about the initiatives in relation to air quality. For example, P8 thought that there was no formal evaluation of School Streets or anti-idling campaigns:

“*I don't think there's any, kind of, strict monitoring and evaluation process in place for the anti-idling work or the School Streets work. I think intuitively, if you're cleaning up the air around the school at times when it's…when we know that it can be bad with drop-offs and pick-ups, that should improve the picture.”* (P8)

P3 specifically noted the additional challenge of collecting baseline air quality data in schools.

Participants reported evaluations using classroom air quality monitors (P1-2, P6, P8, P14-15). Schools were provided with air quality monitors during the COVID pandemic, although it was unclear who provided them (P6, P14), and some schools were part of a specific research project monitoring classroom air quality (P1-2, P8, P15). Some participants mentioned that the monitors raised awareness of air quality (P2, P14). However, schools did not receive feedback on the data collected by the researchers (P2, P15).

Nevertheless, participants shared their views about the potential effectiveness of these initiatives. Some felt that the changes were effective and air quality improved (P4-6, P9, P12-13). For example, when discussed whether the green fencing around the school was working, P2 stated that:

“*Well, we assume the fencing has. We make assumptions that that was done in good faith. It certainly has cut noise pollution down dramatically, so I think that will have worked.”* (P2)

P11, however, did not think the initiatives to encourage active travel would change how children traveled to school as those who lived further away would have to drive anyway. P8 was skeptical of the overall effects as it “*could be moving the problem elsewhere…a little bit further from the school and kids are still walking through lots of traffic.”* Participants also stated that the initiatives could be effective in the short term as they could “*get pupils and parents to start thinking about what they're doing”* (P1), but there was uncertainty about whether they could substantially change things in the long term (P1, P10).

#### 3.3.2 Barriers and facilitators to initiatives

Table 3 provides an outline of the barriers to and facilitators for implementation of air pollution initiatives highlighted in the interviews. Resistance to initiatives from parents was frequently mentioned as a barrier to changes, including refusing to drop off children further from the school, parking on the pavement, idling, always busy and in a rush, having more than one child to drop off at different locations, road and personal safety concerns about letting children travel by themselves (P2-6, P9-10, P12-13, P15). For example

**Table 3:** Barriers to and facilitators for implementation of air pollution initiatives.

**Barriers/ facilitators**	**Description**	**Highlighted in interview (participant reference number)**
Barriers to initiatives	Resistance from parents	P2-6, P9-10, P12-13, P15
	Lack of communication, low awareness and insufficient notification of initiatives	P1
	School teachers too busy	P1
	Insufficient enforcement of campaigns like anti-idling	P7-8, P10
	Initiatives too infrequent to affect change	P1, P5
	Lack of access to bikes in deprived areas	P3
	Theft (of scooters)	P15
Facilitators to initiatives	Parental support and community engagement	P1, P13
	Involvement of children	P3, P6, P8-9, P12-13
	Rewards for children, e.g., badges	P13
	Support from local volunteers and community including charity donations	P4-5, P14
	Community between local environmental health teams and schools	P8
	Media reports	P2
	School leadership boards for initiatives like WOW tracker	P6

WOW, Walk Once a Week.

“*But what we do get as well is parents will come before we close [the road] and they'll sit in the road in their car sometimes with their engine running until they've dropped their children off when the school opens and then they'll drive off. So, they're trying to circumvent what we have in place.” (P4)*

When asked why parents would prefer to drive children to school, P2 stated “*I definitely think one of the issues is the roads being so busy. It's kind of a catch-22, that actually the roads feel quite dangerous.”*

Lack of communication with parents, low awareness or insufficient notification, e.g., closing streets, could result in resistance among parents and residents: “*If parents are unaware of street closures and unexpectedly encounter them, it can lead to backlash.”* (P1)

Participants also mentioned that schoolteachers were already stretched and could not prioritize air quality initiatives (P1). For example, one aspect of the Sustainability, Mental Health, and Environmental Education (SAMHE) project involved offering free indoor air quality monitors to schools, but this initiative saw very little uptake (P1, P8). P1 explained:

“*I can't remember whether SAMHE did have a deadline for kind of expression of interest or not, but generally these things tend to, and a lot of the time they are quite tight turnarounds, and they're not always geared to, okay, well this school has SATS [Standard Assessment Tests] coming up at this time of year, or it has GCSEs [General Certificate of Secondary Education] coming up at this time of year. So if someone's there going, this is great, but ultimately my main focus right now is my pupils' exams, again, it does fall by the wayside*.” (P1)

Issues of prioritization were also reflected in insufficient enforcement [e.g., low penalty for car idling (P7, P10); Department for Environment, Food and Rural Affairs (DEFRA) Burn Better campaign stopped short of banning wood burning (P8)]. The frequency of the initiatives was also deemed inadequate to effect changes (P1, P5). In deprived areas, lack of access to bikes could present as a barrier to cycling initiatives (P3). Theft of scooters was also mentioned once (P15).

Parental support and community engagement were considered essential facilitators for effective initiatives (P1, P13). Involvement of children appeared to be a big facilitator as several participants were pleased with the results when children went to talk to drivers who parked on the pavement or idled (P3, P8, P12) or spoke to their parents about walking or cycling to schools instead of driving (P6, P9, P13). Badges appeared to be an effective way to engage children. For example,

“*…It [WOW tracker competition] was a hit because parents were like, oh my goodness, okay, let's do something, let's get the child much happier.”* (P13)

Support from local volunteers and community, as well as charity donations were important resources for schools (P4, P5, P14). Air quality management in certain areas of Manchester, involving collaboration with local environmental health teams and schools, drove the implementation of initiatives (P8). Media reports and school leaderboards for initiatives, like the WOW tracker, pressured schools to implement changes (P2, P6).

There were initiatives that the schools would like to implement but could not. The challenges they faced were the lack of funding (P1, P4, P10), red tape (P15), lack of information or data (P3) and lack of resources to navigate or implement the initiatives (P1, P5, P8). The physical environment, e.g., restrictions associated with listed buildings (P9), the nature of the site (P15), or inability to reduce traffic of main roads next to the school (P2) were considered insurmountable challenges. The COVID-19 pandemic also stopped some of the initiatives going ahead (P7).

### 3.4 Priorities for future research

Participants mentioned several factors and outcomes they viewed as important to children's health and development. The factors were air quality in and around school (P4, P8, P15), deprivation (P6, P8, P15) or disparities in social background (P2, P6), economic conditions (P6, P8) and geography (P1-2). The role of the COVID-19 pandemic on children's learning development (P2, P11-12) and parenting style, e.g., parents spend lots of time on smartphones (P10), was discussed. Reducing screen time to encourage movement and breaks into lessons to improve concentration was also mentioned (P11).

In terms of outcomes, participants expressed interest in the link between environmental and other factors and learning related outcomes, e.g., cognitive development (P4, P6, P8, P11, P14-P15), SEN (P2, P10, P12-13), neurodiversity (P8, P11, P14), speech (P2, P8), concentration (P11, P14), or retention of knowledge (P11); mental health outcomes, e.g., social, emotional or behavioral issues (P12); and physical health outcomes, e.g., asthma (P8, P12), eczema (P12), allergy (P14). For example,

“*Since COVID, concentration, mental health, social and emotional mental health particularly. There's a lot more issues, we're getting a lot more children with SEN coming in younger years than we ever have had before. We've got 25 per cent of children now with SEN needs, which is a lot.”* (P12).

Participants were interested in how to motivate people to change travel related behaviors (P5). Some participants noted that this was complex and multi-faceted, with cognition, pollution and socioeconomic factors being interconnected (P2, P7-8, P10). P8 emphasized the importance of measuring outcomes related to cognitive functioning, health conditions like asthma, school performance, and attendance, noting that cognitive functioning can be both an outcome and a factor influencing other outcomes like health management or educational attainment:

“*…is there an issue the extent to which a child's cognitive functioning has an impact on the extent to which they're able to use their inhalers, which therefore means that their asthma may not be properly managed, which means they will end up…likely to end up in the health service system.”* (P8)

Participants mentioned that evaluating the impact of air quality initiatives would be challenging, as these efforts might unintentionally worsen social inequalities. For example, P1 raised concerns about initiatives promoting parents walking their children to school, noting that parents in affluent areas are more likely to work from home and participate, unlike those in deprived areas. Furthermore, the complexity of these initiatives lies in their potential to produce both positive and negative health outcomes, often leading to unintended consequences. For example,

“*Things like I suppose, you know inability…the cost of public transport, for example, might mean that a child walks to school rather than gets public transport, which might mean they're exposed to more air pollution. But at the same time, they might be getting more exercise. So, it's how deprivation shows itself rather than deprivation per se I think, which is the thing to think through really.”* (P8)

## 4 Discussion

This qualitative study used primary school settings to explore participants' perceptions about air quality, air-quality-related initiatives in and around schools in GM, deprivation and their relationships with children's health and development outcomes. The interview questions were designed to focus on air quality and related initiatives but allowed room to explore the connections between air quality, pollution, and socio-economic factors. Using a semi-structured interview approach, participants were encouraged to discuss issues they felt were important, even if these topics were not initially identified by the researchers. This open-ended approach led to broadening the scope of findings beyond the initial focus.

While participants acknowledged that environmental factors affect children's general health and development, they perceived the impact of air pollution on conditions like asthma, cognitive development, and SEN to be an understudied area.

Socio-economic influences were identified as a significant contributing factor and a confounding factor to air pollution for children's health and development. This shift from a narrow perspective to a broader, interconnected understanding, highlighted the multi-faceted nature of factors affecting children's health and development. Participants stated that affluent areas in GM often become focal points for environmental activism, advocating for sustainable practices such as active travel to schools. With campaigns promoting walking or cycling as alternatives to driving, these communities are perceived to frequently lead the charge in pushing for air quality improvements, raising awareness and instigating change. However, there's an irony in that these areas are seen as major contributors to local emissions in poorer areas due to their higher levels of consumption and mobility. Our findings align with other research showing that road transport pollution in poorer areas of the UK is largely caused by individuals from more affluent areas (47).

It is worth noting that while walking and car use were the most frequently discussed modes of travel for school drop-offs, public transport, such as buses and trams, was not frequently mentioned. This is likely because primary schools in GM (and the UK in general) are typically within walking distance for most families, particularly in urban areas. However, strengthening public transport services, or introducing a dedicated school bus system, could play an important role in reducing car use for school drop-offs and may represent a valuable public health intervention worth considering in future policy discussions.

The participants felt that people living in more affluent areas tend to have higher rates of working from home and are less reliant on car use for work and school drop offs. Data from the Office for National Statistics (2022–2023) shows that individuals in the highest income bracket, those with degree-level education or higher, and those in professional roles were the most likely to report exclusively working from home or adopting hybrid work arrangements (48). This inequity suggests that the environmental costs of commuting are not evenly distributed across GM, further exacerbating health inequalities.

Additionally, the distribution of green spaces and cycling infrastructure across GM was reported to be unequal. Some participants believed that affluent areas had better access to parks and dedicated cycling paths, promoting healthier lifestyles and active travel. In contrast, lower-income areas were considered to lack these amenities, limiting residents' options for sustainable transportation and recreation. Research has demonstrated significant inequalities in the availability of green spaces, with poorer areas generally having less access compared to wealthier areas (49).

This research highlights that high pollution levels and resource scarcity, like green space, in socio-economically disadvantaged GM areas are major community concerns. It's important that air quality improvement initiatives do not adversely affect these communities. The resulting disparities and unintended consequences have led to divided opinions among participants, emphasizing the intertwined nature of environmental factors and deprivation. This suggests the need for a comprehensive study of these elements together, rather than viewing them as separate influences, to better support initiatives in GM.

The study identified multiple air quality initiatives and problems of evaluation or understanding about their effectiveness, primarily focused on reducing traffic and promoting active travel around primary schools in GM. Initiatives like Living Streets and School Streets were evaluated through platforms such as Modeshift and the WOW digital tracker to monitor trends in mode of travel. However, participants noted that challenges include inaccurate self-reported data and difficulties in data collection due to time constraints and staff motivation. These challenges suggest that while the evaluation platforms have potential, operational difficulties may undermine their effectiveness.

Another gap in knowledge is whether the air quality initiatives in GM have led to measurable improvements in and around schools and children's health and development. Although it is often presumed that initiatives to reduce air pollution near schools, like green screens and road closures, will have direct and measurable benefits. Yet, participants noted that formal evaluation of these impacts is often absent, leaving the true effects open to interpretation. In contrast, evidence from London is more promising: as part of the “Breathe London” project, a study on School Street air quality reported that this initiative can lower NO_2_ levels by up to 23% during school drop-off times (50).

Some schools participated in research projects, including classroom air quality monitoring, but often did not receive feedback on the findings, leaving them uncertain about their effectiveness. A systematic review of community-based participatory research highlights how partnerships between academic institutions and communities have successfully tackled air quality issues (51). The authors highlighted the importance of effective communication and sharing of research findings.

To improve reporting accuracy, classroom surveys and self-reporting travel apps can be paired with observational data or geotagged apps to track children's routes. Sharing research findings effectively is vital for schools to utilize the data in meaningful pollution reduction efforts. Linking air monitoring results with environmental and health outcomes is equally important to help both individuals and the community understand their implications (51). Hence, conducting longitudinal studies to assess effectiveness over time by monitoring changes in travel patterns, air quality, and children's health can help stakeholders grasp the initiatives' long-term impact. Developing a convincing body of evidence around the effectiveness of initiatives will also help to build faith in these activities amongst parents.

Our findings indicate that some parents resist changes, such as dropping children at a distance from school or allowing them to walk independently, due to time constraints caused by managing multiple responsibilities, such as work and multiple school drop-offs, and concerns about road traffic and safety. These findings are consistent with earlier research indicating that a decrease in active travel is influenced by factors such as parental time constraints and safety concerns (52–54). These behaviors reflect a broader challenge in community based public health interventions and encouraging positive behavioral changes. Research emphasizes the importance of consistent communication, direct engagement with parents and educational materials to improve children and parental engagement (55).

Many participants were interested in the link between environmental factors and learning outcomes, such as cognitive development, SEN, neurodiversity, concentration and mental health. With fewer participants interested in the link between environmental issues and physical health such as asthma or allergies.

An important trend noticed by the participants is the rise in children with SEN and cognitive issues entering early years, particularly evident post-COVID. Recent research suggests that increased awareness among educators, healthcare professionals, and parents may have contributed to the rising identification of SEN in children (56). The pandemic period saw a surge in mental health and home learning difficulties in children and young people with SEN, potentially contributing to greater awareness (57, 58). Moreover, in pre-pandemic years, certain neurodevelopmental and mental health conditions may have been underdiagnosed due to societal stigma, limited access to diagnostic services, or a lack of professional training in recognizing early signs (59, 60). These factors likely influenced the trends observed in SEN identification. Participants were trying to make sense of this phenomenon by attributing the surge to changes in parental behavior, reduced socialization opportunities for children during the pandemic, increased parental screen time and using digital methods to teach at school. The finding supports previous research on the impact of lockdowns on children's mental health and behavior (61, 62). Lockdowns also intensified pre-existing lack of support and access to SEN services during the pandemic (63). Furthermore, the negative impacts of smartphone use on children's development and parent-child interactions have been highlighted by existing reviews (64, 65).

This post-COVID phenomenon introduces both challenges and opportunities for research examining the relationship between learning abilities and environmental factors such as pollution. Studies will need to be designed to include varying levels of cognitive development, neurodiversity and learning difficulties among children. This means adapting methodologies to capture how children with these challenges might be differently affected by pollution compared to neurotypical peers. Since pandemic-related factors like screen time, disrupted routines, and reduced socialization are relatively recent, researchers may need long-term studies to understand how they interact with pollution to influence learning over time.

### 4.1 Strengths and limitations

A key strength of our study is a comprehensive exploration of local key stakeholders' views about the intricate relationships among air quality, socio-economic inequalities and primary school children's health and development. We successfully recruited ten schools across GMCA; however, some boroughs were not represented, which may impact the balance of teachers' perceptions in our sample. We consider the geographic focus to be a strength considering the size and socio-economic variability in GM. The sample is relatively small and may not be representative of the views across GM. We did not interview parents but acknowledge that interviewing parents might have provided additional insights into this topic.

It is important to note that some participant responses regarding socio-economic factors may have been influenced by the framing of the questions, potentially introducing some bias, which could be considered as a limitation in interpreting the findings.

### 4.2 Conclusion

This qualitative study explored perceptions of air quality initiatives in and around GM primary schools. It highlights the multifaceted nature of air pollution, socio-economic disparities, and children's health in GM. While barriers such as inequitable resource distribution, data collection challenges, and behavioral resistance hinder progress, targeted policy interventions, improved stakeholder collaboration, and evidence-based strategies can help address these issues.

A post-COVID rise in children with learning and emotional challenges raised concerns about children's cognitive development, presenting both research opportunities and challenges on learning abilities and pollution.

Future research should adopt a holistic and multidisciplinary approach, evaluating environmental, socioeconomic, and behavioral factors alongside a range of outcome measures (related to both health and development), to effectively address the challenges. A strong evidence base of intervention development, including evidence of intervention effectiveness, would help to ensure long-term success of air quality initiatives. Conducting longitudinal studies that track changes in travel behavior, air quality, and children's health outcomes would provide valuable insights for policymakers and community stakeholders.

## Data Availability

The datasets presented in this article are not readily available because this research involved qualitative data generated from our interviews. We then extracted data from these conversations and included it in our article, including quotes. However, the raw qualitative data cannot be shared as it contains identifiable information from the subjects. Requests to access the datasets should be directed to sarah.daniels@manchester.ac.uk.
