# Supplementary material for: Stakeholder perspectives on the effects of environmental and socio-economic factors on children's health and learning: a qualitative study in Greater Manchester, England
Source: Front Public Health. 2025 Jun 18;13:1550439. doi: 10.3389/fpubh.2025.1550439 (PMC12213619; doi:10.3389/fpubh.2025.1550439)
Supplement: Supplementary file 1 [file Data_Sheet_1.docx]

## Supplementary Files

## Supplementary file 1: Consolidated criteria for reporting qualitative studies (COREQ): 32-item checklist

Developed from:

Tong A, Sainsbury P, Craig J. Consolidated criteria for reporting qualitative research (COREQ): a 32-item checklist for interviews and focus groups. *International Journal for Quality in Health Care*. 2007. Volume 19, Number 6: pp. 349 – 357

| **No. Item** | **Guide questions/description** | **Comments** |
| --- | --- | --- |
| **Domain 1: Research team and reﬂexivity** | | |
| *Personal Characteristics* | | |
| 1. Interviewer/facilitator | Which author/s conducted the interview or focus group? | SD, HW and NG conducted semi structured interviews. |
| 2. Credentials | What were the researcher’s credentials? E.g. PhD, MD | SD, HW and NG: PhD |
| 3. Occupation | What was their occupation at the time of the study? | SD and HW: Research Associate  NG: Lecturer in Occupational Health |
| 4. Gender | Was the researcher male or female? | All interviewers were female. |
| 5. Experience and training | What experience or training did the researcher have? | SD, HW and NG had experience with qualitative methods including facilitating focus groups and conducting interviews. |
| *Relationship with participants* | | |
| 6. Relationship established | Was a relationship established prior to study commencement? | Researchers had no professional or ongoing relationship with the participants prior to study commencement. |
| 7. Participant knowledge of the interviewer | What did the participants know about the researcher? e.g. personal goals, reasons for doing the research | Participants were aware that this was research project to explore the impact of air pollution and air quality on children’s health and cognitive development. The research team explained that the aim was to co-design a study to explore the effects that exposure to air pollution and deprivation have on cognitive and academic outcomes for children in GM, and to evaluate the effects of ongoing air pollution interventions on these relationships. |
| 8. Interviewer characteristics | What characteristics were reported about the interviewer/facilitator? e.g. Bias, assumptions, reasons and interests in the research topic | The research team was interested in how the complexities of air pollution and deprivation affect children's cognitive development, as well as in identifying future research priorities from teachers, and council/transport representatives. Prior to this study, NG contributed to a publication about the relationship between TRAP and children’s cognitive development. |
| **Domain 2: study design** | | |
| *Theoretical framework* | | |
| 9. Methodological orientation and Theory | What methodological orientation was stated to underpin the study? e.g. grounded theory, discourse analysis, ethnography, phenomenology, content analysis | The research team conducted thematic analysis.  We analysed the data in an inductive manner (i.e. without preconceived ideas). |
| *Participant selection* | | |
| 10. Sampling | How were participants selected? e.g. purposive, convenience, consecutive, snowball | We used purposive sampling to intentionally select professionals with knowledge, experiences and views of children’s cognitive development and/or air pollution levels or interventions in GM. A snowballing approach was used for some participants. |
| 11. Method of approach | How were participants approached? e.g. face-to-face, telephone, mail, email | Face-to-face, telephone and email. |
| 12. Sample size | How many participants were in the study? | 12 schoolteachers (from 10 schools) and 7 council/transport representatives (from 5 different establishments). |
| 13. Non-participation | How many people refused to participate or dropped out? Reasons? | Of the 21 primary schools that were directly contacted, 10 agreed to participate. We invited 78 representatives from local government and TfGM, of which 7 agreed to participate. We had no response from the 11 schools and 73 local government and TfGM representatives that did not participate. |
| *Setting* | | |
| 14. Setting of data collection | Where was the data collected? e.g. home, clinic, workplace | Interviews were conducted online or at the school premises. |
| 15. Presence of non-participants | Was anyone else present besides the participants and researchers? | There were no people present during the data collection besides participants and researchers. |
| 16. Description of sample | What are the important characteristics of the sample? e.g. demographic data, date | All participants were professional and worked in GM. |
| *Data collection* |  |  |
| 17. Interview guide | Were questions, prompts, guides provided by the authors? Was it pilot tested? | Questions were sent in advance to some TfGM and council participants to enable them to gather information about existing initiatives implemented in GM. |
| 18. Repeat interviews | Were repeat interviews carried out? If yes, how many? | There were no repeat interviews with the same participants. |
| 19. Audio/visual recording | Did the research use audio or visual recording to collect the data? | All interviews were audio recorded with permission of participants. |
| 20. Field notes | Were ﬁeld notes made during and/or after the interview or focus group? | Researchers made field notes during the interviews. |
| 21. Duration | What was the duration of the interviews or focus group? | The interviews had a duration of 30 min to 1 hour. |
| 22. Data saturation | Was data saturation discussed? | Data saturation was reached on all major topics. |
| 23. Transcripts returned | Were transcripts returned to participants for comment and/or correction? | Transcripts were not returned to participants for comment and/or correction. |
| **Domain 3: analysis and ﬁndings** | | |
| *Data analysis* | | |
| 24. Number of data coders | How many data coders coded the data? | Two researchers coded three interviews individually and came together to compose a preliminary coding tree, with feedback from the rest of the research team. The same two researchers used this coding tree to code the remaining interviews independently. |
| 25. Description of the coding tree | Did authors provide a description of the coding tree? | There is no description of the coding tree but can be provided upon request. |
| 26. Derivation of themes | Were themes identiﬁed in advance or derived from the data? | Themes were derived from the data in an inductive manner. |
| 27. Software | What software, if applicable, was used to manage the data? | Researchers used NVivo 14 during the coding of all interviews. |
| 28. Participant checking | Did participants provide feedback on the ﬁndings? | There was no participant check performed by the research team. |
| *Reporting* | | |
| 29. Quotations presented | Were participant quotations presented to illustrate the themes/ﬁndings? Was each quotation identiﬁed? e.g. participant number | Key findings of this study were supported with selected quotes in text. Each quotation was identified by a participant number. |
| 30. Data and ﬁndings consistent | Was there consistency between the data presented and the ﬁndings? | All findings were derived from the data and all themes are supported by illustrative quotes. |
| 31. Clarity of major themes | Were major themes clearly presented in the ﬁndings? | Major themes were derived from the data and are clearly defined by a sub-heading. |
| 32. Clarity of minor themes | Is there a description of diverse cases or discussion of minor themes? | Themes on which there was a deviant view between participants are  discussed. Themes that are only described by one participant are also reported in the results section  of this study. |

Abbreviations: TRAP: traffic-related air pollution; TfGM: Transport for Greater Manchester; GM: Greater Manchester

**Supplementary file 2: Interview questions**

Interview questions for the three key stakeholders: Greater Manchester (GM) primary schools, Transport for Greater Manchester (TfGM) and local councils.

**Questions for primary schools:**

1. **Current/recent air pollution initiatives**

We are trying to gather information of ongoing initiatives about air quality or air pollution in and around schools in GM.

1. Are you aware of any initiatives that are going on or have recently been carried out?

2. If yes, what is the role of your school in these initiatives? If not, move to question 4.

3. What has been your school’s experience with this (these) initiative(s)?

1. What has worked well?
2. What has not worked well?
3. How has your school benefitted from being involved?
4. Do you think these initiatives (quote some answers from Q1) have had some effects?

If yes, in what aspects and to what extent it was effective? What might have contributed to the success?

If no, what might be the reasons?

4. What sources of air pollution are of the most concern to your school?

5. What effects do you think air pollution or environmental factors have on children?

1. Which are you most concerned about in your school?
2. Are any groups of children particularly vulnerable to these effects?
3. (If deprivation or socio-economic disparity did not emerge in answers to 4.b) Research has shown that Air Pollution is higher in deprived urban areas. What is the situation in your area?
4. **Co-design of future study**

We are trying to design a study, which will investigate the relationships between air pollution, air quality and children’s cognitive development (e.g. how quickly and accurately they can process information, and how these skills develop over time). We are also curious about whether socio-economic disadvantage has a role in such relationships.

We’d like to get your input on the design of this study, how it can be of benefit for your school, and how we can make it fit around the normal functioning of primary schools.

6. What do you find interesting about this proposed study?

7. Can you see any other useful outcomes for your school, from taking part in a study like this?

**Questions for TfGM:**

- 1. **Current/recent air pollution initiatives**

We are trying to gather information from ongoing air quality initiatives about local air pollution in and around schools in GM.

1. We understand that TfGM have various ongoing initiatives to mitigate transport–related air pollution in GM. Can you tell us the main initiatives related to schools and children?

2. Do you think these initiatives (quote some answers from Q1) are effective?

3. How are these initiatives being monitored and evaluated?

4. What are the key facilitators of successful actions? What might be the main barriers?

5. What role do you think deprivation (or socio-economic disparity) plays in the effects of air pollution?

**B. Co-design of future study**

We are trying to design a study, which will investigate the relationships between air pollution, air quality and children’s cognitive development. We are also curious about whether socio-economic disadvantage has a role in such relationships.

6. What would be of interest to TfGM about a study in this area of research?

**Questions for local councils:**

- 1. **Current/recent air pollution initiatives**

We are trying to gather information from ongoing air quality initiatives about local air pollution in and around schools in GM.

1. We understand there are various recent and ongoing initiatives to improve air quality in GM and your area. Can you tell us the main initiatives related to schools and children?

2. Do you think these initiatives (quote some answers from Q1) are effective?

3. How are these initiatives being monitored and evaluated?

4. Have you identified successful actions from these initiatives? If so, what factors contributed to the success of these actions? If no, what might be the main barriers to successful actions from these initiatives?

5. What sources of air pollution or environmental factors are of the most concern to children’s health and development in your borough/council/council executive?

6. What role do you think deprivation (or socio-economic disparity) plays in the effects of air pollution on residents in your borough?

**B. Co-design of future study**

We are trying to design a study, which will investigate the relationships between air pollution, air quality and children’s cognitive development. We are also curious about whether socio-economic disadvantage has a role in such relationships.

7. What would be of interest to your council about a study in this area of research?

8. What do you think are the key aspects we need to consider when doing this kind of research?

Supplementary file 3

Coding structure for thematic data analysis. Code names in **bold** represent main themes, while code names in regular text indicate sub-themes.

| Code Name |
| --- |
| **Current challenges** |
| Attendance |
| Learning ability |
| Mental health |
| Physical health |
| Safety |
| **Factors (affect outcomes)** |
| Air Pollution not concerned  *(not concerned about AP in local area or not considering it as a contributing factor for the challenges)* |
| Environmental factors |
| Other factors |
| Socio-economic factors |
| **Future research** |
| Design suggestions |
| Expected output |
| Feasibility |
| Interested factors or outcome |
| Suggestions for engagement |
| Barrier |
| Facilitator |
| **Initiatives** |
| Barriers (for initiatives) |
| Challenges for implementation |
| Effectiveness (of the initiatives) |
| Evaluation (of the initiatives) |
| Facilitators (for initiatives) |
| Ongoing initiatives |
| Other |
| School environment |
| Travel |
| **Local environment (descriptive)** |
| Physical environment |
| Social demographic |
| **Role of key stakeholders** |
| Local Authority |
| Transport for Greater Manchester |
